# Antecedents of trust and customer loyalty in online shopping: The moderating effects of online shopping experience and e-shopping spending

**DOI:** 10.1016/j.heliyon.2023.e16182

**Published:** 2023-05-10

**Authors:** Thabang Excellent Mofokeng

**Affiliations:** Department of Marketing Management, School of Consumer Intelligence and Information Systems, College of Business and Economics, University of Johannesburg, South Africa

**Keywords:** Customer trust, Customer loyalty, On-time delivery, Online shopping experience, e-shopping spending

## Abstract

This paper determines the antecedents of trust (i.e., perceived ease of use [PEoU], privacy concerns [PC], perceived security [PS], product variety [PV], and on-time delivery [OD]) and customer loyalty (CL) in online retailing. A questionnaire was developed using scales validated in prior e-commerce studies to measure these factors in the conceptual model. Data were collected in an online survey from a non-probability judgement sample of online shoppers between the ages of 18 and 65 years old, who provided informed consent for participation in the survey. Data were analysed via structural equation modeling (SEM) on AMOS version 28. The ethical approval certificate was issued by the College of Business and Economics Research Ethics Committee (CBEREC). The results indicate that customer trust (CT) in online shopping relies on OD, PS, PV, and PEoU, but not PC. CT, followed by OD and PV, significantly impacts CL. The results show that trust mediates the relationship between OD, PS, and PV, and CL. Online shopping experience and e-shopping spending significantly moderate the impact of PV on trust. The impact of OD on CL is significantly moderated by the online shopping experience. This paper validates a scientific approach to coexisting effects of these key forces that e-retailer practitioners can use to gain trust and build CL. Research that validates this valuable knowledge is absent in the literature, as the factors were measured disjointedly in prior studies. This study offers originality by validating these forces in online retailing in South Africa.

## 1 Introduction

With the expansion of diverse interconnected devices, e-retailers, such as Amazon.com and Alibaba, integrate artificial intelligence (AI) technologies to present innovative products and services, deliver smart consumer services, analyse consumer behaviour, and optimise logistics. This has transformed the methods of online purchases and sales [1,2]. With the Internet of Things (IoT), Amazon uses a smart-speaker device integrated into various smart-home compatible devices and its website connects to the ecosystem, which enables consumers to use voice commands to purchase products and services from the comfort of their homes, which delivers a unified online shopping experience [2]. These innovations in online shopping have transformed consumers' behaviour from passive browsers of web pages to active interactions and interactive conversations with e-retailers. Due to a lack of face-to-face interactive communication, including the ability to touch the products, consumers perceive risks in e-commerce [3]. From these innovative web pages, the research question is whether the online shopping experiences that e-retailers offer impact perceived ease of use (PEoU), privacy concerns (PC), perceived security (PS), product variety (PV), and on-time delivery (OD) as antecedents of customer trust (CT), and whether these factors build customer loyalty (CL). Specifically, whether factors such as PS, PC, PV, and OD build customers’ preference for online shopping over traditional shopping [4] and help to acquire and retain online consumers [5].

This study aims to examine a unified model comprising all the above-mentioned online retailing forces using a non-probability judgemental sample of online consumers in an emerging economy setting and testing it using structural equation model (SEM), as recommended by Ref. [6]. Scholars have measured the impact of dimensions comprising PEoU [7], PC [8], PS [9], PV [10], and the pay-on-delivery mode of payment [11] on CT. However, such studies presented disjointed technical frameworks of determinants that measured and validated unique forces and their significant effect on CT and CL in the e-commerce setting. In examining this knowledge gap, this study aims to validate the combined significance of these factors in a single model. Currently, it is not known which of these cognitive forces affect the growth of online shopping, in general. While studies examined the antecedents of and obstacles to online retailing growth as e-commerce system concepts, other key factors of trust such as PV and OD in online shopping remain unexamined. Furthermore, comprehensive secondary data to validate these factors in emerging economies are lacking, as most studies originated in developed countries [[7], [8], [9]]. To date, an inclusive model that conceptualises PEoU, PC, PS, PV, and OD as the online retailing forces that influence CT and CL, mediated by trust and moderated by online shopping experience and e-shopping spending, has not been developed. It is relevant to measure these gaps in the literature. Therefore, this study sought to validate a unified model that conceptualises all these key variables in a South Africa's online shopping context. Firstly, this unified framework will contribute to knowledge that e-retailers and policymakers can utilise to establish CT and build CL when customers perceive these factors in online retailing within emerging market settings with huge growing potential. Secondly, the paper contributes empirical evidence on how to generate trust in online retailing as a mediator of consumers' perceptions of websites ease of use, privacy, security, PV, and OD, on CL. Thirdly, validating the moderating effects of online shopping experience and e-shopping spending builds insights to marketing theory in understanding consumer behaviour and developing market profiling in the online retailing. Understanding the effect of these forces on CT and how to implement them to build CL will increase online retail sales in emerging economies. Lastly, this knowledge contributes to the technology acceptance theory, marketing philosophy, and strategic retailing planning for profitable future business models.

The conceptual model in this study was built on the premise that customers’ usage of computer technologies is increased by the main factor of PEoU [12]. Despite technology being useful and interactive, customers develop negative attitudes if they perceive it as complex or risky [11]. In addition, it would be worthwhile to assess the impact of PS by consumers concerning the handling of their personal data, as one of the indubitably interesting constructs, on the quality of the relationship [13]. Research points out the need to identify and address the tension between information use and PC [14]. [15] also recommended the research to measure a PV and countries as e-retailers need to hold a consistent balance in the actual in-store product variety and the information provided online.

Interestingly, while a great variety of quality products affect consumers’ purchase behaviour from online stores [16,17], the possible direct significant impact of PV on CT has not been validated in business-to-consumer (B2C) e-commerce [18]. found variety, value for money, and delivery as important attributes for online shoppers. However, prior studies have not validated the influence of PV on CT in the presence of PEoU, PS, PC, and OD. This concept is not traditional in the literature of marketing and is therefore not clearly conceptualised [19]. [20] show the distinct variances of variety seeking, product involvement, impulsiveness, etc., and consumer-directed segmentation as important factors in the relationship marketing literature. In e-commerce [21], insist that scholars should examine the variety of products available and their price as other factors that might affect the formation of e-loyalty. Based on these studies, it is evident that the inclusion of measures of PV and CT in developing e-loyalty is needed. E-retailers who compete on wide PV and the efficiency of the logistical or delivery operations can gain trust and develop consumer loyalty in the South African setting. Research that empirically validates the unified impact of these variables is scant.

As customers' private data in the online retailing marketplaces are transferred to external logistics service providers to ship the products to the customers [22], the more parties are involved, the higher consumers' concerns over control on who can accesses their private data (i.e. home or work address, preferences, and payment details) [23]. Vendor perceived risk refers to the extent to which consumers believe that they could incur losses, such as the problems of payment methods, disclosure of personal information, delivery of products, etc. when purchasing from the Internet vendors [8,24]. [11] show a significant impact of pay on delivery mode of payment on customer trust. Research in high-uncertainty-avoidance societies [25], and high uncertainty avoidance cultures [26] shows cash on delivery as an important factor that positively impact customer trust. Hence, removing the key obstacles of online retailing growth, such as (1) technological errors that reduce perceived value, (2) problems to secure payment and build consumers’ confidence in disclosing credit card data, and (3) the fulfilment of cheaper logistics and quick delivery [27], is a key path to its success.

In South Africa [28], notes that the logistical factors of goods in the country are common problems in online shopping. After the customer has paid, it might take a long time to receive the product due to the product packing, shipping, and delivery process. The product could be damaged prior to its arrival, which leads to returning it to the seller, and the customer is liable for the return costs. Fulfilment (e.g., delivery timeliness, order accuracy, and delivery conditions) plays an important role in perceived overall e-service quality [10], as an e-retailer's website fulfilment/reliability influences e-satisfaction and e-trust [29]. The pay-on-delivery mode of payment builds trust among online shoppers in emerging markets like India, where e-retailers need to spread product deliveries across highly distant geographical areas [11]. Further [10], found that customers who prefer cash-on-delivery payments and transfer methods of bank payments in Indonesia worried less about their card data security. Thus, incorporating measures of PV and OD in the model shows how e-retailers in emerging markets can compete on these factors in regular practice. Managing consumers' evaluations of these services might help to grow online shopping in the emerging market context. This study's approach contributes a strategy path for e-retailers to gain CT and build CL to their websites [[30], [31], [32]]. [33] showed that trust, relative to customer satisfaction, is a key determinant of e-customer loyalty in small and medium-sized enterprises (SMEs) in the Kingdom of Saudi Arabia.

In addition to Ref. [34], who measured the moderating effect of experience on the determinants of PEoU [35], note the moderating effect of user experience, and suggest factors such as PS as an important and useful angle for future research. Scholars have measured the moderating effects of online shopping experience on the relationship between customer satisfaction and repurchase intention [36]. Others [37], found a positive relationship between Internet experiences and online shopping frequency, which lean on the view that consumers who spent many years using the Internet are likely to engage more in online shopping [38]. insist that it is necessary to assess more issues, such as the average cost of online shopping (per month) or product categories of purchase, in order to provide a more comprehensive evaluation of consumers' online shopping behaviour. It is unknown whether and how online shopping experience and e-shopping spending moderate the interactions between online retailing forces, CT, and the CL behaviour of South Africans. The literature has not validated the moderating effects of these variables on the precursors of CT and CL in the online retailing setting. Interestingly, the global retail market was expected to reach $27 trillion in 2022 [39], and it is projected that the revenue in the e-commerce market in the Southern Africa region would reach $8.09 billion in 2023. This revenue is estimated to reach an annual growth rate (CAGR 2023–2027) of 12.66%, accounting for a market volume of $13.03 billion, as the number of users in the e-commerce market will reach 41.40 million by 2027. While the market volume of $1487.00 billion is expected in 2023, majority of this revenue will derive from China [40]. The McKinsey Global Institute projected that e-commerce will reach $75 billion by 2025 in South Africa, as Africa's leading economy [41].

Based on the predictable growth of e-commerce in South Africa and the introduction of innovative web pages in online retailing, this study aimed to examine the valuable implication of trust for online retailing by validating a unified theoretical framework that measures the key antecedents (e.g., PEoU, PC, PS, PV, and OD) of trust in online retailing. The model determines the direct impact of CT, PV, and OD on CL, in addition to validating the mediation of trust [7,42,43], and moderating effects of online shopping experience [36] and e-shopping spending [37]. This research ideation contributes to the existing body of knowledge on how online trust guides technology acceptance theory, marketing philosophy, science, and practice.

The study follows the following structure: Section 2 outlines an understanding of the theoretical basis of this study. The literature review and development of the hypotheses are discussed in Section 3. Section 4 explains the research methodology used in this study. The data-analysis methods and results are presented in Section 5, while Section 6 discusses the results of the study and links them to theoretical and managerial implications. Section 7 concludes the paper by discussing the study's limitations and providing directions for future research.

## 2 Theoretical background

Academics have validated models aimed at the adoption and acceptance of information technology. This study adopted the technology acceptance theory to measure the impact of PEoU, PC, PS, PV, and OD on CT, and the direct effects of PV and OD on CL. Online trust mediates the relationship between PEoU, PC, PS, PV, OD, and CL, moderated by online shopping experience and e-shopping spending in online retailing setting.

### 2.1 Technology acceptance model (TAM)

**Fig. 1:**
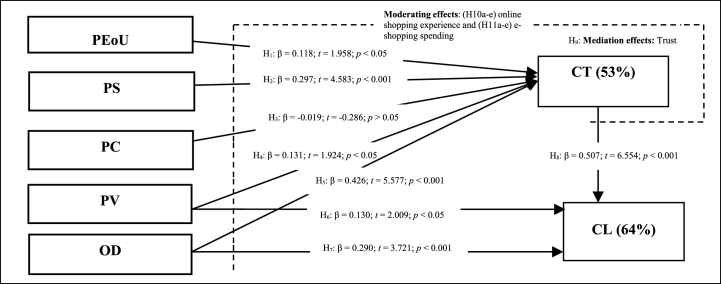
Theoretical framework for measuring antecedents of CT and CL in online retailing: The moderating effects of e-commerce experience and e-shopping spending.

The TAM was developed by Ref. [12] to explain the effects of PEoU and perceived usefulness of technology systems on users' diffusion and adoption. Perceived usefulness refers to “the degree of the person's beliefs that the use of a particular system improves her or his job performance”, while PEoU refers to “the degree of the person's beliefs that the use of a particular system requires less effort” [12]. The TAM maintains strong theoretical and explanatory power in current literature on user acceptance and adoption of new technology [9,44,45]. Accordingly, the TAM was developed from the Theory of Reasoned Action (TRA) proposed by Ref. [46] to explain that intention will best predict a person's behaviour. The mediation of intention on the relationship between PEoU, perceived usefulness, and usage behaviour was confirmed by Ref. [47]. The main modification between the TAM and TRA is that the TAM measures two elements of technology acceptance, namely perceived usefulness and PEoU, while the TRA captures the component of consumer attitude in the development of the intention [12]. Grounded in the TAM and prior research on CT [48], found information quality, security concerns, PEoU, perceived usefulness, and PC as the five main drivers of CT to repeatedly use social media in the Kingdom of Saudi Arabia [49]. found that PEoU and perceived usefulness remain the two most relevant antecedents of trust toward online travel websites in Egypt (a culture that is high in uncertainty avoidance). The importance of CT, PS, and PC of users of online trading systems was studied by Ref. [50] using the TAM [51]. applied the Service Quality (SERVQUAL) model and the TAM, coupled with proposed quality factors, in relation to m-commerce that, according to the literature, influence CT. The TAM is viewed as appropriate for evaluating consumers' attitude to the ease of use of online shopping websites [5,52]. Research shows the positive effect of PEoU on customer attitudes toward online shopping [53]. Some evidence reveal that PEoU relative to the usefulness of technology quality is the strongest predictor of online customer satisfaction [51,54], perceived website trust [49], the customer's behavioural intention to adopt Internet shopping [5,33], and convenient motivation [55]. Clearly, PEoU is a key feature of customers' usage of online technologies [12]. This evidence supports the conceptual model (see Fig. 1) that measured the effect of PEoU (while usefulness is assumed to be represented by other factors in the framework), on CT in online retailing.

According to Refs. [42,43], trusting intentions will naturally be influenced by trusting beliefs because the TRA posits that beliefs influence intentions. These authors developed a typology of trust types showing that trust beliefs and trust intentions in e-retailers partially mediate the impact of web-based retailer interventions on consumer behaviours [7]. studied the interaction between the TAM and trust in online retailing. They found that the intentions of experienced consumers to repurchase from e-retailers rely on trust, including the two perceptions related to the TAM, namely PEoU and perception of usefulness [56]. verified this hypothesis in the courier services context. Prior studies thus reported the need for e-retailers to establish trust in Internet transactions by improving PEoU [49,57,58]. Notwithstanding the evidence showing a strong direct positive impact of perceived usefulness on attitude towards using mobile apps [59], information systems (IS) continuance intention [60,61], technology acceptance in accounting studies [62], consumers' intention to use online food delivery services [63], and use mobile payment [64], this study adopted the TAM as an appropriate model to assess the role of PEoU in building consumer trust in the online retailing setting. Perceived usefulness is a dependent variable on PEoU, suggesting that PU is predicted by PEoU [12]. Research shows perceived ease of use as significant and positive factor that impact perceived usefulness and customers’ usage intentions toward technology acceptance [65]. In addition to the constructs of the TAM [9], showed that earning trust and monitoring processes of security are key beliefs that induce behavioural intention.

The marketing literature emphasises brand trust and its role in developing brand loyalty [20,66], and relationship commitment [67], but the importance of online trust generated by PEoU, PC, PS, PV, and OD as key elements of e-commerce CL remains unclear. According to Ref. [33], it is important to identify factors that play a significant role as predictors of perceived trust in e-commerce stores. It is recommended that future studies examine e-trust and its components [68]. Establishing a trusted website is a key issue for brands as consumers tend to buy from and stay loyal only to websites that they trust [30]. Trust is a key asset that enhances participation in e-commerce in general, especially in an online retailing setting due to the greater ease for e-retailers to practise opportunistic behaviour [7]. Trust in online retailing poses many problems and contains specificities that cannot be ignored, such as a lack of face-to-face interactive communication, and the ability to touch the product, which increase uncertainty and risk perception in consumer's online purchasing decision [8]. Establishing CT is a continuous and dynamic practice that relies on regular interactions [42,43], as customers gain trust in a website based on their interactions with it, and the level that it stimulates positive perceptions [70]. In general, earning trust in electronic marketplaces is regarded among the main objectives in online retailing, e-shopping, e-marketing, etc. [69].

In summary, trust is a strategic factor in the process of information exchange and for establishing relationships in online retailing [1,24,71]. A relationship built on trust in the e-retailer and brand loyalty are drivers for buyers to choose higher-priced products [2]. Due to the lack of previous experience, users will perceive great uncertainty and risk when they adopt e-commerce for the first time, and they need to build initial trust to overcome perceived risk [72]. New users at first doubt purchasing online [16], while experienced Internet users shop frequently from a website they perceive as fun, easy to ease, and convenient [5]. The TAM lean on knowledge of Internet usage, and aid the testing of the online shopping experience and e-shopping spending as moderating factors in the model.

## 3 Literature review and the development of hypotheses

A conceptual model was developed based on the findings in the literature (see Fig. 1) and tested for validity using SEM in AMOS version 28, with the aim to contribute new knowledge on online retailing growth in South Africa. The model hypotheses the direct influence of each independent variable on the dependent variables. PEoU, PC, PS, PV, and OD are theorised as elements of CT. Online trust mediates the relationship between PEoU, PC, PS, PV, OD, and CL. In addition, CT, PV, and OD are theorised as determinants of CL in online retailing. Lastly, online shopping experience and e-shopping spending moderate the theorised causal structure.

### 3.1 Perceived ease of use (PEoU)

PEoU refers to “the person's perception to find the use of a particular system to relieve him or her from mental and physical effort” [12]. Ease of use indicates the level of non-complexity, which refers to a perception of effortless use of Internet interfaces [45]. This shows the need “of solving the complexity by designing of interfaces offering users […] easy-to-understand” options [73]. Because consumers avoid trying an innovation unless a strong link builds trust in that innovation, the degree of ease of using a website and its utilities can establish CT. Research shows that PEoU influences consumer attitude in online purchases [53], it has a significant effect on e-commerce adoption [33], and it contributes positively to building CT in online shopping [7,58]. This study therefore hypothesised the following.

H1 Increased PEoU positively and significantly impacts CT in a website.

### 3.2 Perceived security (PS)

Security in B2C e-commerce is explained in terms of technologies that protect and safeguard consumer data [74]. The level of sensitivity in functioning and activities that consumers perform at the stage of transactions makes the direct influence of security on online adoption a primary concern, which is often viewed as a main reason that customers avoid online purchases [9,75]. [76] believe the considered threat to e-commerce adoption is not personal data security per se, but a consumer's perception thereof [13]. suggest that it is necessary to examine and plan the correct measures to resolve consumers' concern for the security of personal data in the Internet setting. Customers who perceive an online transaction as not safe and secure will prioritise shopping from traditional stores [16]. PS refers to “the safety and protection consumers perceive about credit card data in the online transactions” [70]. Studies on online purchase intention in high-uncertainty-avoidance societies [25], and high uncertainty avoidance cultures [26] note problems pertaining to security, privacy, and trust as important reasons that impact customers' uncertainty related to adopting e-payment systems (i.e., debit/credit cards) and electronic commerce. It is vital to examine the PS of online transactions and credit cards by online customers in developing countries such as South Africa with a culture that is high in uncertainty avoidance (UA), often viewed as a major barrier on the adoption of technology [77]. Due to increased fear of data theft or fraudulent credit card purchases, shoppers in emerging markets prefer cash payments over using credit/debit cards [11,28,44]. Marketers must include website security as a strategy to compete and achieve e-commerce success in emerging markets. Studies on PS have empirically validated this construct interchangeably with privacy [10,31,32]. A good understanding of the role of security in growing online retailing is needed. This study examined the PS of websites that emerges when and if security mechanisms that an e-retailer offers, such as encryption, authentication, etc., fail to protect an online transaction [1]. Offering these security features increases trust, and the consumers' perception of good security and trust drive the adoption of e-commerce usage [74,78]. In e-marketing, designing websites with secure online transactions builds CT [69]. This study therefore hypothesised the following.

H2 Increased PS positively and significantly impacts CT in a website.

### 3.3 Privacy concerns (PC)

Privacy refers to “the safety of identifiable person's information on the Internet, and comprises the acceptance and execution of a notice, disclosure, consent of users, and privacy policy on the website” [70]. The sensitivity of customers about their personal data has increased, and they feel offended and engage in avoidance behaviour towards an e-retailer that violates their privacy [79]. Often, users prefer privacy while browsing websites, and a higher level of privacy in online shopping and the absence of consumer control of personal data increase the perception of risk [1]. Online privacy is linked to problems such as security, fraud, and trust barriers that slow down e-commerce growth [1]. Trust in information privacy is very important for customers [22]. Despite good recognition of the association between privacy and trust in the literature [8,70,79], it is relevant to examine customer trust in privacy assurances linked to technology (e.g., encryption) as opposed to vendor-related measures (i.e., privacy seal and policy) as the main questions are aimed at unveiling insights into privacy and trust matters [1]. Trust, together with customers' experience with information systems, can impact the intention to disclose personal information [73], as the trust in protecting personal data is higher for buyers with less PC [81]. On the other hand, consumers who have greater concerns about and valuation of privacy place greater emphasis on privacy violations in forming trust judgements [82]. Hence, PC negatively relate to attitudes towards online shopping [83], they have a negative impact on trust in e-commerce [8], and they have shown a negative impact on consumers' trust in using social media for e-retailing services [48]. This study therefore hypothesised the following.

H3 Increased perception of PC negatively and significantly impacts CT in a website.

### 3.4 Product variety (PV)

Two perspectives describe PV, namely the quantity of offers and the variety of offers [84]. Because of the potential reach of the Internet and its ability to help search for services and specialty goods, customers expect a broad PV from e-retailers [85]. More consumers shift their buying activities from traditional stores to online stores and the driving force of this transformation in purchase behaviour is to acquire the benefits that e-retailers offer, such as easy access to a wide variety of product categories, improved product diversity and service selection, greater convenience, lower prices, and time saving [16]. Offering a product portfolio that matches the market niche and responding accurately with products that meet customers’ needs increase satisfaction, develop CL, inspire positive word-of-mouth communication, stimulate repeat sales, and offer other cross-selling benefits [86]. [17] found that consumers in China prefer PV when the level of heterogeneity is high. Perceived PV in an online store is viewed as an important factor that affects consumer behaviour [17,87,88], but it is unclear whether PV influences CT in e-commerce. This is another unique contribution of this study. A monopoly retailer operating an online store (often referred to as the channel owner) can offer PV, sell products directly to consumers, and aid many other independent manufacturers to use its website to sell products [17]. These stakeholders, including customers, may have varied perceptions of and desires for e-retailer trust [80]. Furthermore, when a product is obtainable exclusively at one store, consumers can visit only that store to buy the product, as opposed to when a product is available at multiple stores because they could easily switch e-stores [88]. This study therefore hypothesised the following.

H4 Increased PV perception positively and significantly impacts CT in a website.

H5 Increased PV perception positively and significantly impacts CL to a website.

### 3.5 On-time delivery (OD)

On-time delivery (OD), is a dimension of fulfilment/reliability referred to by Ref. [89] as “the accurate display and description of a product so that what customers receive is what they thought they ordered, and (b) delivery of the right product within the time frame promised”. It is regarded as the capability of a retailer's website to deliver the exact product in the expected condition and at the expected time. Despite the noticeable e-commerce growth, consumers fear that the promised products will not conform to their expected levels and that the demanded products will not be delivered by the e-retailer [3]. From the customer's perspective, time delivery is an additional important factor that may generate a perception of unreliability towards the website of an e-retailer, especially if the e-retailer repeatedly delays the delivery schedules [29]. Delivery can be flawed by late delivery, or non-delivery risks, including unforeseen changes in e-retailers’ promises [69]. Perceived risk reflects consumers' concerns relating to the selling party's ability to not fulfil formal and informal responsibilities; for example, breaking promises in terms of delivery, privacy policy, or product quality levels [24]. [37] examined the different effects that final delivery solutions may have on online shopping. E-retailers must reduce the delivery time to avoid the chance of consumer regret. In situations where a lengthier delivery time is unavoidable, the buyer must be informed about the estimated arrival time of the ordered goods. Providing real-time delivery information will help consumers make smarter collection choices. The unique benefits of products purchased should also be explained to shoppers [90]. Many online customers prefer direct-to-home delivery [37] and expect reliable and on-time delivery [88]. This is similar to the marketing literature [66,67] positing trust as existing when one party has confidence in the integrity and reliability of an exchange partner. Research shows that pay-on-delivery mode of payment [11], cash on delivery [25,26], and fulfilment/reliability (i.e. time delivery) [29] have a positive impact on CT [86]. found that delivery features strongly impact on CL. This study therefore hypothesised the following.

H6 Increased OD perception positively and significantly impacts CT in a website.

H7 Increased OD perception positively and significantly impacts CL to a website.

### 3.6 Customer trust (CT) and customer loyalty (CL)

CL is a construct studied broadly in the research on online retailing. It is measured extensively in theoretical frameworks that explore the adoption of technology. Strategies to instil trust can foster CL in online shopping, which will result in improved consumer relationships and increased profits for the e-retailer in the long term [31]. This study measured CL as the concluding dependent variable in the conceptual model [20]. note that loyalty, which leads to repeat purchase behaviour, is generated by trust. Greater levels of trust entail a positive customer attitude towards an e-retailer's website [31]. Trust in B2C e-commerce is a necessary determinant of CL. Empirical evidence shows that CT has a positive significant impact on CL [[30], [31], [32]]. In general, the level of e-commerce participation has been growing, which can be attributed to increasing interest in the concept of trust [11,71], which shows that online shopping growth relies on customer trust in e-retailers [44]. In addition to research measuring the mediation of trust in online shopping behaviour [7,42,43], there is evidence showing that CT partially mediates the relationship between website features and behavioural intent more strongly for some website categories than for others [70]. This study therefore hypothesised the following.

H8 Greater levels of CT positively and significantly impact CL to a website.

H9 Greater levels of CT in an online shopping website mediate the relationship between e-commerce platform attributes (a: PEoU, b: PS, c: PC, d: OD, and e: PV) and e-commerce CL.

### 3.7 Online shopping experience and e-shopping spending

Customer experience denotes the overall experience customers have with a retailer, including interactions with and beliefs about its brand(s) [91]. The latest innovative technologies (i.e. automation, machine learning, etc.) in online retailing also offer greater potential for improving customers' experience by helping e-retailers evaluate customers' preferences and behavioural shopping patterns [91]. Online consumers expect a more convenient and happier online shopping experience [37]. Extending the research by Refs. [36,92], of specific interest to this study is the moderating role of online shopping experience, which is defined as “the volume of purchases that a customer has completed in the past” over the Internet [71]. measured customers' web experience as length in the number of years a customer used the Internet. Customers who acquire more Internet experience and e-commerce usage have lower PC [1], establish trust in a website, and generate trust in online retailing [71]. The Internet experience may influence customers’ purchase intention [3] and build CL [31,93] in online retailing. Another important factor is e-shopping spending, which is measured by expenditure on online shopping and its diffusion rate in total shopping consumption [37]. In extending the work done [36,37], and [92], this study examines the moderating effects of online shopping experience and e-shopping spending when customers perceive PEoU, PS, PC, OD, and PV as key attributes developing CL behaviour with minimum trust. This study therefore hypothesised the following.

H10 Online shopping experience moderates the influence of e-commerce platform attributes (a: PEoU, b: PS, c: PC, d: OD, and e: PV), CT, and CL to the extent that e-commerce platform attributes influence CT and CL less strongly when online shopping experience is higher.

H11 E-shopping spending moderates the influence of e-commerce platform attributes (a: PEoU, b: PS, c: PC, d: OD, and e: PV), CT, and CL to the extent that e-commerce platform attributes influence CT and CL less strongly when e-shopping spending is higher.

## 4 Research methodology

### 4.1 Instrument design

This study used a quantitative research method. The validated multi-item scales were adapted from prior studies to design the instrument capturing four scale items of PEoU [75], three scale items of PC [94], and five scale items of CT [95]. OD, PV [96], PS, and CL [86] were assessed with four scale items each. All 28 scale items adapted in this study (see Table 1) (except for demographic data) measured responses using a five-point Likert scale ranging from (1) strongly disagree to (5) strongly agree. These items, written in English, were adjusted towards the main objective of this study. The wording of the items was refined and sequenced, tested for logical consistencies, and the instructions and level of comprehension were made clear, and using professional-quality layout. The online version of the questionnaire was designed and website link of the pilot test was posted on a research company's website (*n* = 50) to examine all the challenges that the respondents may encounter in the reading and completion of the questionnaire in online survey. The potential respondents were referred to this website link to participate and requested their feedback. The values of Cronbach alpha ranged from 0.632 on PV to 0.887 on PS. Pilot test improved the final version of the questionnaire and the overall quality of the survey. The content validity and face validity of the instrument verified its accuracy for use in the initial online survey, as it takes little effort to complete.

**Table 1:** Scale items and their source.

Construct	Item	β	α	CR	AVE
**Perceived ease of use (PEoU)** [75]					
It is easy to place an order on this website.	PEoU^1^	0.724	0.827	0.829	0.619
*It is easy to shop on this website.*	PEoU^2^	–	–		
It is easy to learn the shopping procedure on the website.	PEoU^3^	0.811			
Everyone can easily master the shopping procedure on this website.	PEoU^4^	0.822			
**Privacy concerns (PC)** [94]					
The website clearly explains how user information is used.	PC^1^	0.739	0.747	0.753	0.507
Only the personal information necessary for the transaction to be completed needs to be provided.	PC^2^	0.591			
Information regarding the privacy policy is clearly provided.	PC^3^	0.791			
**Perceived security (PS)** [86]					
The website has security precautions to ensure secure payments.	PS^1^	0.731	0.889	0.882	0.654
The website has the contents of the data protection declaration.	PS^2^	0.762			
I feel I can trust the guarantees offered by this website.	PS^3^	0.871			
This website adheres to compliance with data protection rules.	PS^4^	0.861			
**Product variety (PV)** [96]					
The product range of this website is complete.	PV^1^	0.717	0.724	0.763	0.518
*The products I get from this website can be found in other similar websites.*	PV^2^	–			
I can easily find products that I need on this website.	PV^3^	0.678			
There are more choices for goods of a particular type on this website.	PV^4^	0.761			
**On-time delivery (OD)** [96]					
The product is delivered by the time promised by the company.	OD^1^	0.709	0.800	0.814	0.593
*I get what I ordered from this website.*	OD^2^	–			
The items sent by the website are well packaged and perfectly sound.	OD^3^	0.779			
I am satisfied with the delivery mode of the website (post, express delivery, home delivery).	OD^4^	0.819			
**Customer trust (CT)** [95]					
This e-commerce vendor is trustworthy.	CT^1^	0.785	0.878	0.884	0.607
This e-commerce vendor provides reliable information.	CT^2^	0.605			
This e-commerce vendor keeps promises and commitments.	CT^3^	0.838			
This e-commerce vendor keeps my best interests in mind.	CT^4^	0.884			
This e-commerce vendor's behaviour meets my expectations.	CT^5^	0.756			
**Customer loyalty (CL)** [86]					
I will recommend the online shop to my friends.	CL^1^	0.800	0.842	0.842	0.573
I intend to visit the online shop in the future.	CL^2^	0.766			
I intend to shop at the online store in the future.	CL^3^	0.796			
I will also buy other products from online shops in the future.	CL^4^	0.658			

Note: Constructs' reliability and validity [β = standardised factor loading; α = Cronbach's alpha; AVE = average variance extracted].

### 4.2 Data collection

Data were collected through a research company's website using an online survey involving online consumers in South Africa. The South African population of online customers, both male and female respondents between the ages of 18 and 65 years old, who were shopping online during the month of the survey, participated in the study, which represents the country's population. It is worth noting that a large portion of the South African population (i.e. an estimated 15.6 million individuals by 2020) resides in the Gauteng province [97]. Potential respondents were asked to recall their recent online shopping experience and refer to this experience when answering the questionnaire. A non-probability sampling method of judgement criteria of selecting online shoppers was used, which deliberately question those who are consuming online shopping services, as the person who has the most knowledge and experience was questioned [98]. The screening process ensured the inclusion of only respondents who purchase from web stores (i.e., online vendors and/or retailers' websites). For this screening, they were asked: Are you shopping from web stores, and how long have you been using web stores to purchase products and services online? This judgement criteria was aimed to elicit the ability of the respondents to participate in the study, and sought to select only consumers whose online shopping experience exceeded six months [98]. The study was conducted after obtaining the ethical approval certificate from the College of Business and Economics Research Ethics Committee (CBEREC). Marketing research ethics principles (e.g., protect anonymity and privacy of the participants and the confidentiality of the data provided) were followed. A questionnaire was distributed for self-administering by online shoppers who provided informed consent for voluntary participation in the study. An Internet Protocol (IP) address assigned to the website registered the respondents and helped to avoid duplication in data entry. This also enabled sending an e-mail to specific respondents to thank them for their participation.

From a total of 300 male and female respondents between the ages of 18 and 65 years old who participated in this study, 285 pre-coded questionnaires (or a 95% response rate) contained valid responses of customers' perceptions of online retailing services in South Africa and were processed (i.e., tabulated) into the Statistical Package for the Social Sciences (SPSS) version 28 and AMOS version 28 for analysis. Categorical data from the consumer profiles show that 38.6% of the respondents were male and 61.4% were female; 17.2% were under 24 years old, 27% were between 25 and 29 years old, 29.1% were 55–59 years old, and 17.2% were above 60 years old; 35.4% of the respondents reported spending R5 000 to R6 000 monthly on online shopping; 30.2% of the respondents had six to 12 months' online shopping experience, 26.7% exceeded one year experience, and 28.8% had less than three years’ experience; most respondents purchase on Takealot.com (37.9%), followed by Spree.com (16.1%) and Amazon.com (13%); and 46.0% of online consumers bought clothing and 33.7% bought electronic gadgets. These results show that the online shoppers had good knowledge and experience to partake in the study.

## 5 Analysis and results

*Common method of bias*: The priori and post-hoc research procedures for eliminating the risk impact of common method bias in the dataset were conducted. At a priori level, the questionnaire was developed using validated scale-items from the previously studies, and was piloted [99]. All the respondent were assured of their anonymity in participation and confidentiality of data provided. At a post hoc level, the common method bias was tested, as per the recommendations in scientific research [99,100], using Harman's single-factor test using Exploratory Factor Analysis (EFA) on SPSS version 28, whereby all of the 28 items measuring each construct in this study were captured to load on one single factor. The results showed that the factor extracted 31.71% of the variance, which is less than the 40% threshold [101], which shows that measurements were unlikely to suffer from common method bias issues. In confirming the results, the study estimated measurement properties of Confirmatory Factor Analysis (CFA) model using AMOS version 28 which loaded all items into a single factor. The model fit of the one-factor model (χ^2^ = 857.193, df = 525, χ2/df = 1.633, standardised root mean square residual (SRMR) = 0.039, goodness-of-fit index (GFI) = 0.858, comparative fit index (CFI) = 0.931, root mean square error of approximation (RMSEA) = 0.047) is significantly worse (Δχ^2^ = 478.125, Δdf = 247, p < 0.001) than that of the hypothesised CFA model (χ^2^ = 379.068, df = 251, χ2/df = 1.510, SRMR = 0.033, GFI = 0.909, CFI = 0.964, RMSEA = 0.042). This confirms the absence of risk effect of CMB in the data collected and analysed in this study, i.e. data is not polluted by CMB.

### 5.1 Testing the measurement model

A confirmatory factor analysis (CFA), using maximum likelihood estimates, analysed the psychometric properties of all inter-related constructs in the model (see Table 1 for the factors' reliability and validity). The measurement model drew covariance-based matrix input into AMOS version 28 to measure the 28 items of the following elements: PEoU, PS, PC, PV, OD, CT, and CL. The CFA verified if any outliers existed in the model, such as Heywood cases or negative error variances, including standardised factor loadings higher than 1.0 or lower than −1.0 [98]. The factor loadings exceeded 0.70, except for PC2 = 0.59, PV3 = 0.68, CT2 = 0.60, and CL4 = 0.66, which showed relatively low convergent validity. Despite items with factor loadings <0.5 being considered for removal [102], a factor loading of 0.59 was retained because its value was very close to 0.6, also see Ref. [103]. In general, factor loadings and CR should be equal to or greater than 0.7 for good convergent validity [104]. The standardised residuals below 2 in absolute value established the correctness of the model [105]. The measurement model had adequate fit indices ([χ^2^ (df), p = 379.068/251 = 1.510, p < 0.001, SRMR = 0.033, GFI = 0.909, adjusted goodness-of-fit index (AGFI) = 0.882, Tucker–Lewis index (TLI) = 0.957, CFI = 0.964, non-normed fit index (NFI) = 0.901, incremental fit index (IFI) = 0.964, and RMSEA = 0.042]) [106]. Table 1 shows the Composite Reliability (ρ) and Cronbach's alpha (α) testing of the reliability of the constructs, while the convergent validity and discriminant validity confirmed the appropriateness of the measurement model [107]. The accurate reliability of a construct is naturally captured by Cronbach's alpha and composite reliability (CR), but Cronbach's alpha unweights items, which makes it a less precise measure of reliability, while CR weights the items built on factor indicators' matrix loadings, which result in the Cronbach's alpha being lower than the CR [107]. All the factors had Cronbach's alpha values above 0.7, and the CR values were also above 0.7 [104]. Factors with values closer to 1 (i.e., PV = 0.674 and deleted PV^2^ = 0.724, PC = 0.747, PEoU = 0.809 and deleted PEoU^2^ = 0.827, OD = 0.839, CL = 0.842, CT = 0.878, and PS = 0.889) in Table 1 shows internal consistent reliability. The CR (ρ) values (PC = 0.753, PV = 0.763, OD = 0.814, PEoU = 0.829, CL = 0.842, PS = 0.882, and CT = 0.884) were above 0.7, as recommended by Ref. [104]. All the variables in Table 1 had average variance extracted (AVE) values exceeding >0.5, which showed the convergent validity of the scales measured [107].

Discriminant validity was confirmed when the scale items per factor converged on their own true scores, which showed factor identity matrices from the other constructs. Each construct had the square root of the AVE exceeding its interaction with the other variables in the scale (see Table 2), which showed the discriminant validity of the factor correlations as below 0.7 [104,107]. To define the necessary sample size [104], suggested that scholars should be dependent on power analyses that reflect their model structure, the expected significance level, and the anticipated effect sizes. These authors insist on the use of ten repetitions for the indicator means from the analysis sample. As seen in Table 1, with 28 items measured in this study, a sample of 300 was deemed appropriate.

**Table 2:** Discriminant validity of the factor correlations.

Construct	CL	PS	OD	PV	CT	PEoU	PC
CL	**0.757**						
PS	0.436***	**0.809**					
OD	0.674***	0.411***	**0.770**				
PV	0.499***	0.298***	0.467***	**0.719**			
CT	0.753***	0.543***	0.638***	0.458***	**0.779**		
PEoU	0.307***	0.342***	0.318***	0.383***	0.402***	**0.787**	
PC	0.359***	0.425***	0.423***	0.321***	0.359***	0.252***	**0.712**

Note: The values of the square root of the AVE are bold in the diagonal line, while inter-construct correlations are in the off-diagonal matrix. p < 0.001***.

The direct and indirect specified hypotheses in the conceptual model were tested and validated via structural model (SEM) with AMOS version 28 (maximum likelihood estimation) in the next section. SEM is a two-step model technique, useful in simultaneously testing the psychometric measurement properties and the theorised paths to validate the structural model; thus enabling researchers to examine the psychometric properties of the adopted scales and their resulting underlying relations [105]. This analytical technique is ideal for testing and confirming theory by developing a covariance-based SEM that analyses the goodness-of-fit indices (GFIs), unlike the less rigorous variance-based partial least squares technique [104], and it also generates latent variable scores for analysis of mediation and moderation. The PROCESS Procedure for SPSS Release 2.041, with the Johnson-Neyman conditioning method [108], tested the mediating and moderating variables at a 95% level of confidence.

### 5.2 Testing the structural model

The structural model input in AMOS version 28 (see Fig. 1) was tested using maximum likelihood estimates [105]. The results of the fit indices show an adequate model fit ([χ^2^/df = 379.893/254 = 1.496, p < 0.001, RMR = 0.034, GFI = 0.909, AGFI = 0.884, CFI = 0.964, IFI = 0.965, TLI = 0.958, NFI = 0.901, and RMSEA = 0.042]) [106]. The R^2^ values of CT was 53%, and CL was 64%, and both >50%.

### 5.3 Direct effects, mediation, and moderation effects

Based on the literature, the direct effects proposed in the structural model (see Fig. 1) specified that PEoU (H_1_), PS (H_2_), PV (H_4_), and OD (H_6_) had a positive direct influence on CT, while PC (H_3_) had a negative significant impact on CT. Additionally, the model indicated that PV (H_5_), PD (H_7_), and CT (H_8_) had a positive direct impact on CL. CT (H_9_) mediated the relationship between independent variables and customer loyalty. Online shopping experience (H_10a-e_) and e-shopping spending (H_11a-e_) significantly moderated the hypothesised structured-model. The bootstrap analytical method using maximum likelihood tested the bias of modified confidence interludes.

The findings in Fig. 1 and Table 3 reveal that PEoU (β1 = 0.118, p < 0.05), PS (β2 = 0.297, p < 0.001), PV (β4 = 0.131, p < 0.05), and OD (β5 = 0.426, p < 0.001) had a positive direct influence on CT in online retailing. PC (β3 = −0.019, p > 0.05) had insignificant negative impact on CT, thus rejecting H_3._ The direct effects proposed support H_1_, H_2_, H_4_, and H_5_. This shows that consumer services such as OD, PS, PV, and PEoU contribute positively to establishing CT in a website. Furthermore, the results show that PV (β6 = 0.130, p < 0.05), OD (β7 = 0.290, p < 0.001), and CT (β8 = 0.507, p < 0.001) have a positive direct impact on CL towards a website. The direct effects proposed in H_6_, H_7_, and H_8_ were validated. CT, OD, and PV contribute positively to developing CL. Fig. 1 shows that 53% of the variance in CT is explained by OD, PS, PV, and PEoU, while 64% of the variance in CL is explained by CT, OD, and PV.

**Table 3:** Summary of the direct effects.

H	Hypotheses	β	*t*(>1.96)	*p*	Result
H_1_	PEoU positively and significantly impacts CT.	0.118	1.958	0.050	Accepted
H_2_	PS positively and significantly impacts CT.	0.297	4.583	0.001	Accepted
H_3_	PC negatively and significantly impact CT.	−0.019	−0.286	0.775	Rejected
H_4_	PV positively and significantly impacts CT.	0.131	1.924	0.054	Accepted
H_5_	OD positively and significantly impacts CT.	0.426	5.577	0.001	Accepted
H_6_	PV positively and significantly impacts CL.	0.130	2.009	0.045	Accepted
H_7_	OD positively and significantly impacts CL.	0.290	3.721	0.001	Accepted
H_8_	CT positively and significantly impacts CL.	0.507	6.554	0.001	Accepted

Note: [*p* *<* 0.001***; *p* *<* 0.01**; *p* *<* 0.05*].

Mediation analysis of e-commerce trust was conducted through the bootstrapping procedure for total, direct, and indirect effects developed by Ref. [108] and implemented through Model 4 of PROCESS Procedure for SPSS Release 2.041. The results of direct and indirect effects in Table 4 were obtained using 1000 bootstrap samples at 95% level of confidence. Table 4 shows the findings of the mediation analysis of trust in the structural model.

**Table 4:** Direct and indirect effects.

H1–H8: Direct effects					
Predictor	CT				
	Effect	*t*_*(>1.96)*_	*p*	LLCI (95%)	ULCI (95%)
					
					
					
PEoU	0.1241	1.7646	0.0792	−0.0146	0.2628
PS	0.0647	5.8945	0.0000	0.2406	0.4825
OD	0.3812	5.5567	0.0000	0.2459	0.5165
PV	0.2454	3.3273	0.0010	0.1000	0.3909
H9: Indirect effects					
Predictor	Mediator	Effect	*p*	BLLCI	BULCI
PEoU	CT	0.3399	0.0000	0.2198	0.4808
PS	CT	0.2969	0.0000	0.2204	0.4048
OD	CT	0.3342	0.0000	0.2400	0.4610
PV	CT	0.3093	0.0000	0.1757	0.4940

Note: ***: p < 0.001, **: p < 0.01, *: p < 0.05. [Low-Level Confidence Interval - LLCI, Upper-Level Confidence Interval - ULCI, Boot Lower Level Confidence Interval - BLLCI, and Boot Upper-Level Confidence Interval - BULCI].

H_9_ proposes that CT mediates the relationship between (a: PEoU, b: PS, c: PC, d: OD, and e: PV) and e-commerce CL. PS, OD, and PV, except PEoU (β = 0.1241, p > 0.0792), had significant direct impact on customer loyalty. The study examined the indirect effects of (a: PEoU, b: PS, c: PC, d: OD, and e: PV) on CL through CT. The results in Table 4 confirm the significant indirect effect of PEoU on CL (β = 0.3399, p < 0.001) through CT, the significant indirect effect of PS on CL (β = 0.2969, p < 0.001) through CT, the significant indirect effect of OD on CL (β = 0.3342, p < 0.001) through CT, and the significant indirect effect of PV on CL (β = 0.3093, p < 0.001) through CT. It can be practical that the relationship between (a: PEoU, b: PS, d: OD, and e: PV) and CL is indirectly affected by CT in online retailing. H_9a_, H_9b_: H_9d_ and H_9e_ are accepted, while H_9c_ is rejected.

Moderation analysis of online shopping experience and e-shopping spending was conducted through the bootstrapping procedure for indirect effects developed by Ref. [108] and implemented through Model 1 of PROCESS Procedure for SPSS Release 2.041. The results were obtained using 5000 bootstrap samples at a 95% level of confidence. Table 5 indicates the findings on the moderating effects of online shopping experience and e-shopping spending in the structural model.

**Table 5:** The moderating effects of online shopping experience and e-shopping spending.

Model 1 PV (X) and CT (Y) experience (M)	Coeff	BootMean	BootSE	*t*	*p*	BootLLCI	BootULCI
Constant	−0.2279	−0.2720	0.8447	−0.3000	0.7645	−1.9742	1.3433
Variety	1.0410	1.0512	0.1994	5.6489	0.0000	0.6591	1.4474
Experience	1.1219	1.1416	0.3044	3.5788	0.0004	0.5397	1.7445
Int_1	−0.2699	−0.2745	0.0723	−3.5275	0.0005	−0.4171	−0.1311
Conditional indirect effects PV (X) and CT (Y) Experience (M)							
Experience	Effect	se	*t*	*p*	LLCI	ULCI	R^2^
Low	0.7711	0.1200	6.4286	0.0000	0.5346	1.0077	0.1874
Medium	0.5012	0.0808	6.2057	0.0000	0.3419	0.6605	
High	0.2313	0.1018	2.2717	0.0242	0.0305	0.4321	
Model 2 OD (X) and CL (Y) experience (M)	Coeff	BootMean	BootSE	*t*	*p*	BootLLCI	BootULCI
Constant	−0.2065	−0.1672	0.6096	−0.3935	0.6943	−1.3290	1.1248
Deliver	0.9582	0.9491	0.1377	7.6287	0.0000	0.6628	1.2084
Experience	0.5354	0.5281	0.2343	2.4796	0.0140	0.0754	0.9956
Int_1	−0.1122	−0.1105	0.0535	−2.1527	0.0326	−0.2173	−0.0050
Conditional indirect effects OD (X) and CL (Y) Experience (M)							
Experience	Effect	se	*t*	*p*	LLCI	ULCI	R^2^
Low	0.8460	0.0829	10.2088	0.0000	0.6826	1.0094	0.4515
Medium	0.7338	0.0582	12.6020	0.0000	0.6190	0.8486	
High	0.6216	0.0731	8.5020	0.0000	0.4774	0.7658	
Model 3 PV (X) and CT (Y) e-spending (M)	Coeff	BootMean	BootSE	*t*	*p*	BootLLCI	BootULCI
Constant	0.8049	0.8079	0.7546	1.1750	0.2414	−0.6664	2.2622
Variety	0.7935	0.7932	0.1790	4.7877	0.0000	0.4474	1.1483
Spending	0.4226	0.4273	0.1641	2.3740	0.0186	0.1141	0.7572
Int_1	−0.1010	−0.1023	0.0392	−2.3608	0.0192	−0.1814	−0.0266
Spending	Effect	se	*t*	*p*	LLCI	ULCI	R^2^
Low	0.6925	0.1301	5.3218	0.0000	0.4359	0.9491	0.1584
Medium	0.4905	0.0827	5.9299	0.0000	0.3274	0.6536	
High	0.1875	0.1384	1.3549	0.1770	−0.0854	0.4605	

Note: *Model 1* product variety: PV (X) and customer trust: CT (Y); experience (M); *Model 2* on-time delivery: OD (X) and customer loyalty: CL (Y); experience (M); and *Model 3* product variety: PV (X) and customer trust: CT (Y); e-spending (M). ***: p < 0.001, **: p < 0.01, *: p < 0.05; SE: standard error). [Low-Level Confidence Interval – LLCI; Upper-Level Confidence Interval - ULCI].

**Fig. 2:**
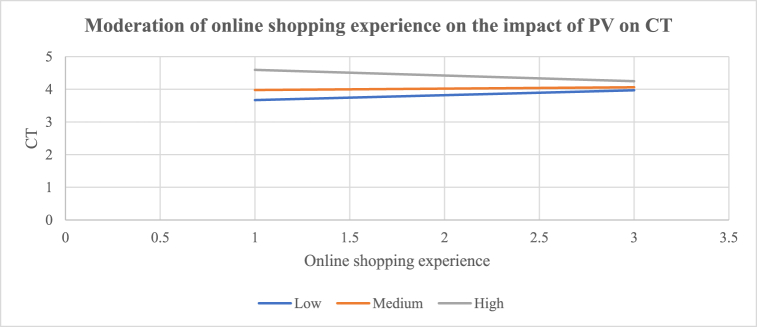
Moderating effect of e-commerce experience on the impact of PV on CT [H_10e_: β = −0.2699, t = −3.5275, p < 0.0005) (R^2^ = 0.1874; p < 0.0001); BLLCI = −0.4171; BULCI = −0.1311].

**Fig. 3:**
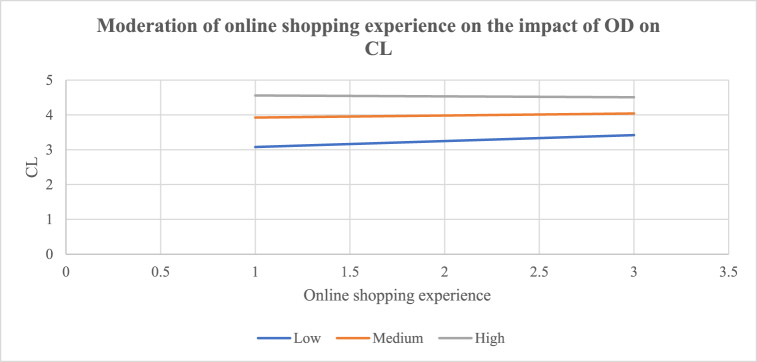
Moderating effect of e-commerce experience on the impact of OD on CL [H_10d_ = −0.1122, t = −2.1527, p < 0.0326) (R^2^ = 0.451; p < 0.0001); BLLCI = −0.2173; BULCI = −0.0050].

**Fig. 4:**
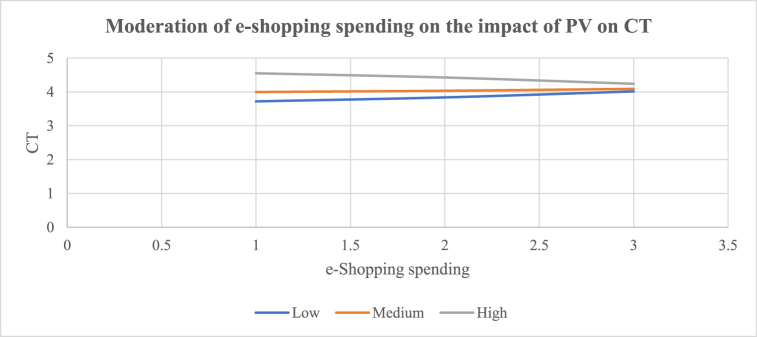
Moderating effect of e-shopping spending on the impact of PV on CT [H_11e_ = 0.1010, t = −2.3608, p < 0.0192) (R^2^ = 0.1584; p < 0.0001); BLLCI = −0.1814; BULCI = −0.0266].

H_10_ proposed that online shopping experience moderated the impact of e-commerce platform attributes (a: PEoU, b: PS, c: PC, d: OD, and e: PV), CT, and CL to the extent that e-commerce platform attributes influence CT and CL less strongly when online shopping experience is higher. The results show that online shopping experience has a negative significant interaction effect between PV and CT [(β10e = −0.2699, t = −3.5275, p < 0.0005) (R^2^ = 0.1874; p < 0.0001)]. The results show that online shopping experience has a negative significant effect on the relationship between OD and CL [(β10d = −0.1122, t = −2.1527, p < 0.0326) (R^2^ = 0.4515; p < 0.0001)]. H_10e_ and H_10d_ are thus accepted. The observed relationships between PV and CT, and OD and CL were negatively and significantly moderated by online shopping experience. H_11_ proposed that e-shopping spending moderated the influence of e-commerce platform attributes (a: PEoU, b: PS, c: PC, d: OD, and e: PV), CT, and CL to the extent that e-commerce platform attributes influence CT and CL less strongly when e-shopping spending is higher. The results show that e-shopping spending has a negative significant effect on the relationship between PV and CT [(β11e = −0.1010, t = −2.3608, p < 0.0192) (R^2^ = 0.1584; p < 0.0001)]. H_11e_ is therefore accepted. It shows that the impact of PV on CT is negatively and significantly moderated by e-shopping spending. The results of the conditional effects (i.e., high or low) of the moderators are shown in Table 5. Fig. 2 shows that the interaction between PV and CT (β = 0.7711; t = 6.4286; p < 0.0000) was strongly significant for customers with low online shopping experience (LLCI = 0.5346; ULCI = 1.0077), than for customers with high online shopping experience (β = 0.2313; t = 2.2717; p < 0.0242) (LLCI = 0.0305; ULCI = 0.4321). Online shopping experience contributed [R^2^ = 0.1874] 18.74% of the variance of the effect of PV on CT. Fig. 3 shows that the impact of OD on CL (β = 0.8460; t = 10.2088; p < 0.0000) was more strongly significant for customers with low online shopping experience (LLCI = 0.6826; ULCI = 1.0094) than for customers with high online shopping experience (β = 0.6216; t = 8.5020; p < 0.0000) (LLCI = 0.4774; ULCI = 0.7658). Online shopping experience contributed [R^2^ = 0.4515] 45.15% of the variance of the effect of OD on CL. Fig. 4 shows that the effect of PV on CT (β = 0.6925; t = 5.3218; p < 0.0001) was significant for customers with low e-shopping spending (LLCI = 0.4359; ULCI = 0.9491) but not for customers with high e-shopping spending (β = 00.1875; t = 1.3549; p < 0.1770) (LLCI = −0.0854; ULCI = 0.4605). E-shopping spending contributed [R^2^ = 0.1584] 15.84% of the variance on the effect of PV on CT.

## 6 Discussions and conclusions

The findings from this study suggest that OD, PS, PV, and PEoU of websites are the key antecedents of trust in online retailing. The results indicate the direct influence of CT, PV, and OD on CL. CT indirectly mediates the significant relationships between PEoU, PS, PV, and OD, and CL. Online shopping experience [36] and e-shopping spending [37] negatively moderated CT, PV, OD and CL interactions. This contributes valuable insights to online retail planning, strategy, and allocation of resources to the cost-effective features of CT in online shopping in the South African setting, and accelerates e-commerce growth. Forty-six percent (46%) of customers purchasing clothing and 33.7% purchasing electronic gadgets are the largest market segments in the South African online retailing sector, while 37.9% of customers purchase from Takealot.com, 16.1% purchase from Spree.com, and 13% purchase from Amazon.com.

In replying to the research question this study examined, OD, PS, PV, and PEoU of websites were found as the key antecedents of CT in online retailing. OD (i.e., delivery time, schedule, etc.) is the strongest element of CT to purchase from a website. The body of knowledge in the subject area shows a significant impact of delivery on customer satisfaction [37,86,90]. However, these studies have failed to recognize the role of OD as a strong prerequisite for customers to trust online retailing, which contributes to the literature [11]. found that the pay-on-delivery mode of payment significantly and positively enhances trust. In expanding this knowledge, the results of this study show a positive significant impact of OD on CL, which contributes a new perspective. It shows that rational customers desire OD, which strongly builds trust and loyalty in online retailing in emerging economies such as South Africa. OD was followed by the significant positive effect of PS on CT. Compared with other researchers who could not show a significant effect of PS risk on e-customer trust in Pakistan [109], there was a pronounced positive effect of PS on CT in South Africa, which supports the literature [9,48,110]. This shows the sensitivity of consumers about the security of online shopping websites before they establish trust in e-retailers in developing countries such as South Africa. Acquiring cybersecurity skills on new technologies to defend top threats from a risk of being hacked and protect online transactions can help e-retailers to increase PS of their websites and build trust as a competitive advantage. Consistently updating the security of the website with innovative tools (e.g., socket-layers, encryption) enhances customer trust. PS was followed by PV in instilling CT. PV on online shopping websites has a positive significant effect on CT, which is another unique contribution to the literature. The literature only shown the impact of product attributes on customer satisfaction [84,86,96], yet satisfaction strengthens CL when their levels of trust in the e-business are higher [30]. Furthermore, a direct positive and significant impact of PV on CL was confirmed by Ref. [111], who examined assortment (the variety of products or services a website offers), which had no direct effect on CT, but had a significant effect on CL [86]. could not find a significant impact of product portfolio on CL. As a unique contribution of this study, it shows that customers establish trust and develop loyalty to an e-retailer that offers PV. Providing PV on a website removes the issue of customers' repeat visits without buying any product, as the depth of product variety on a retailer's website significantly and positively enhances CT and CL. It shows that alternative available substitutes may establish trust in a website. As customers expect a broad PV from e-retailers [85], offering a wide product variety might establish trust and encourage repeat sales especially when e-retailer uses exclusive distribution strategy (i.e. one web store sell the product). In addition, products of channel members such as independent manufacturers who use retailers' website can acquire sales.

Somewhat surprisingly, the results show that PEoU followed PV in significantly and positively affecting CT [49]. found PEoU as the strongest predictor of perceived website trust in travel websites, which contradicts the results of this study, which show the lowest standard loading of PEoU among other significant factors. Notwithstanding its weak impact, this result is similar to the findings by Ref. [112], which showed a weak relationship between ease of use and online trust. Due to the proliferation of technologies recently introduced in the South African online retailing sector, e-retailers’ websites are perceived by consumers as easy to use. It shows how today's consumers are familiar with digital devices and technology uses. While prior studies could not confirm PEoU as a significant predictor of CT on the Internet [113], in e-commerce settings [114] and in mobile shopping context [115], this study supports the literature that shows how PEoU minimises the efforts needed to purchase, which builds CT in online retailing [7,58]. The significant effect of websites' PEoU on CT has been identified in various technology settings such as social media for e-retail services [48], travel websites [49], online trading systems [50], courier services [56], and m-commerce [57]. Therefore, this study supports the strong explanatory power of the TAM's dimension of PEoU of websites in electronic exchange relations [12].

It is questionable on how privacy concerns may exert impact on customer trust in e-commerce. Quite surprisingly, while negative effect of PC on e-commerce trust was found, but there was no evidence of the statistical significance of this effect. Possibly, consumers in South Africa do not see privacy as an antecedent of their trust. Alternatively, it may be the case that South African online shoppers believe that e-retailers adhere to the Protection of Personal Information Act (POPI Act), and Consumer Protection Act. This is similar to findings by Ref. [50], which showed an insignificant effect of PC on CT in online trading websites [116]. found the low relevance of website privacy in the determination of e-service quality among the three services, e.g., online travel reservations, accommodation reservations, and online ticketing, as surprising too. This result fails to support e-commerce literature [8,117] that shows a negative significant influence of PC on CT [82,118]. confirmed that consumers who have greater concerns about and valuation of privacy place greater emphasis on privacy violations in forming trust beliefs [119]. showed that privacy strongly relates with brand trust.

Despite the study that showed convenience, delivery, and time saving as antecedents of satisfaction and the most important reasons for buying online in South Africa [28], the findings of this study reveal that online shoppers in South Africa view OD, PV, PS, and PEoU as better key antecedents of trust in online shopping than the display of privacy policy statements on websites. Possibly a study aiming on technology privacy assurances (e.g., encryption) rather than typical e-retailer-based measures (e.g., privacy policy) is needed [1] to assess and determine its impact on CT. Research studies suggest that PS, compared to PC, has a strong positive effect on CT in e-commence [78,120], as users generally understand the concept of security better than privacy, and security is a more concrete concept. People rate objects they understand as important [74].

The results contribute to the literature by validating CT in online shopping as a strong determinant of CL [[30], [31], [32]]. Together perceptions of OD, PS, PV, and PEoU instil CT. CT, OD, and PV build CL. An e-retailer providing OD and PV on its website meets diverse customers' needs, which develops CL in the online retailing setting. CT indirectly influences the relationship between PEoU, OD, PV, PS, and CL. In a study measuring repurchase intentions relative to customer loyalty [121], showed that CT mediates the relationship between PEoU, PS, and customers’ repurchase intention. CT also influenced the relationship between PC and repurchase intention, which the current study could not validate.

While [122] found that the number of years of online shopping (online shopping experience) is negatively related to PEoU and PS, the results of this study showed that online shopping experience negatively and significantly moderated the influence of PV on CT, including the impact of OD on CL to the extent that the positive impact of perceived OD and PV on CT is more important for customers with low online shopping experience. Furthermore [122], report that the amount spent on online shopping is negatively related to PEoU and PS. This study thus showed that e-shopping spending negatively and significantly moderated the relationship between PV and CT to the extent that perception of PV influences trust in the e-retailer only for customers with low e-shopping spending. It shows the important roles of online shopping experience and e-shopping spending in moderating the impact that OD and PV have on consumer behaviour in e-commerce. As OD and PV are important, in markets where it is hard to earn trust due to the higher PC, e-retailers must provide OD and PV as new customers spend little to gain online shopping experience via website interactions. Providing on-time delivery services is a profound marketing strategy that can stimulate consumer e-shopping spending [37].

Generally, OD, PS, PV, and PEoU are positive significant forces affecting CT in online retailing and developing CL within an empirically tested model. The uniqueness of these results unveil the theoretical and managerial implications for researchers and online retail practitioners. Business and society can learn how the OD, PS, PV, and PEoU can help the online retail growth strategy in transforming online shopping experience and increasing e-shopping spending in the emerging markets and growing the success of B2C e-commerce. No other research study has validated the unique combined effect of these online consumer services, CT, and CL. Knowing how online shopping experience and e-shopping spending moderate these behavioural perceptions offers valuable insights by merging the technology acceptance theory, marketing philosophy, and online retail strategy for future profitable e-business models within an emerging market setting such as South Africa.

### 6.1 Theoretical implications

This study contributes new insights into the literature on online retailing. It highlights the role of TAM [12] used in this study. Firstly, the study validated the importance of additional variables of OD, PS, PV, together with PEoU as important dimensions of TAM that predict CT in online retailing. Adding OD, PS, and PV on this theory help to understand a wide perspective of B2C e-commerce. The results in this study offer e-commerce practitioners insights on how the theoretical framework could advance this theory-based knowledge and show how this theory, when applied in online shopping, could consider the relevant contribution of these factors in e-retailing.

The study has contributed to the understanding of this framework or theoretical foundation in many ways. While studies have examined CL and its role in building successful relationships with customers [[30], [31], [32]], no other study has validated OD and PV as resourceful relationship techniques to enhance CT and CL in online retailing. Measuring these customer services contributes to a better understanding of their significant role, especially OD as a strong significant force that instils CT in online retailing [26]. found that cash on delivery has a positive effect on CT, and that timely product delivery positively impacts customer satisfaction. These authors did not assess the direct impact of timely product delivery on CT. However, they recommended future studies to explore the impact of the moderating variable such as experience.

Together with CT, OD and PV build CL in the context of online retailing. The findings expand the research by Ref. [86], and [111] which showed the significant effects that a product portfolio and delivery have on the satisfaction and loyalty of shoppers in online retailing. Since the opportunity to touch the product lacks in online shopping [3], delivering the expected product on time is a key strategy for building e-commerce CT. Importantly, customers in online shopping are uncertain about whether the product ordered will be delivered. It is necessary for an e-retailer practitioner to ensure the correct delivery time and communicate in situations of delays, which can strongly build CT and develop CL. Communicating delivery time information decreases uncertainty and develops CT [123].

Similar to results in previous studies [84,86], experts in e-retailing could improve merchandise assortment levels for online shopping. Adjusting PV expands alternative available sets of items in online retailing and increases customers’ purchasing options when the desired product is not readily available. The approach to offer PV is appropriate to reduce the perceived risk when high-involvement products reduce purchase intentions [50,70].

A valuable contribution of this study is the validation of OD, PS alongside PV, including PEoU, as key factors that generate CT in online retailing. The results suggest that e-retailers can consider this conceptualisation of the study. This study validates the importance of PS of websites in conducting transactions in the online shopping context of South Africa. It originated in validating PS as a strong generator of CT in the e-retailer following OD. Previous research also identified several factors in the way that they influence customer trust in e-commerce. It was found that experts when encountering the problem, first they can improve customers’ perception of security of their websites. Due to a lack of secured e-payment systems, and increased fear of data theft or fraudulent credit card purchases, shoppers in emerging markets prefer cash on delivery payments over using credit/debit cards [11,25,26,28,44]. This study addresses the need to examine and understand the PS of online transactions and credit cards by online customers in developing countries such as South Africa with a culture that is high in uncertainty avoidance (UA), often viewed as a major barrier on the adoption of technology [77]. This study adopted the TAM as an appropriate model to validate the role of PS in building consumer trust in the online retailing setting. This followed [9] who showed that in addition to the constructs of the TAM, earning trust and monitoring processes of security are key beliefs that induce behavioural intention.

The results of this study, however, did not describe the statistical significant negative impact of PC on CT. This suggests that online customers appeared to place less emphasis on the privacy of their information they disclose to the e-retailers’ websites and were unable to perceive risks related to its use by unauthorised third parties in the transaction. Based on the findings, PC were not significant predictors of CT, as expected. This appears to be a limit on e-retailers’ knowledge in this study. Research reported a negative effect of privacy concerns on trust in e-commerce [8], including social media uses in e-retailing services [48].

This study contributes better insights from the technology acceptance theory, through argumentative and empirical evidence of online retailing forces that establish trust in the minds of customers and develop loyalty [7]. found that the intentions of experienced consumers to repurchase from e-retailers rely on trust, influenced by a perception related to the TAM construct, namely PEoU. Similar to previous studies, e-retail practitioners in this study can establish trust in Internet transactions by improving PEoU [49,57,58], which was also reported in the courier services context [56]. Prior shopping online, customers in this study consider and evaluate OD, PS, and PV that e-retailers offer, and whether a website is easy to use. They establish trust in e-retailers who provide OD, secure online payments, and offer PV on their websites that are easy to use. In addition towards building trust, an e-retailer should offer OD and PV, which enhance CL. Knowledge of this strategic online retailing plan contributes new understanding to the e-commerce literature.

Similar to the results in previous studies, this study contributes understanding that validates the strong association between CT and CL in online retailing. Research in B2C e-commerce [30,31] and m-commerce [115] shows the influence that CT has on CL, but the current study is the first to validate OD and PV on websites as key strategic relationship techniques that can concurrently affect CT and build CL. Together with CT, the significant impact of OD and PV may successfully develop CL to e-retailers in the e-retailing setting.

The study has contributed to the understanding of the framework of trust in e-commerce. Based on the TRA [42,43], showed that trusting beliefs influence trusting intentions. Their typology of trust types shows that trust beliefs and trust intentions in e-retailers partially mediate the impact of web-based retailer interventions on consumer behaviours. Another important contribution of this study is that the relationships between PEoU, PS, OD, PV, and CL are positively and indirectly mediated by CT in online retailing [70]. showed that CT partially mediates the relationship between website features and behavioural intent more strongly for some website types than for others. Incorporating these website cues can enhance CT and CL, thus forming a long-term favourable consumer relationship with the firm, and trust cues need to be explicitly incorporated into website design strategies.

Extending the work done by Refs. [36,37,92], the results of this study validate the moderating effects of online shopping experience and e-shopping spending when customers perceive OD and PV to build CL behaviour with minimum trust. Results show the relevance of OD and PV as variables that reinforce the trust and loyalty of customers with low online shopping experience and low e-shopping spending in the South African setting [93]. reveal that the traditional marketing mix and shopping experience of the Japanese are strong indicators of their e-satisfaction and e-trust. They insisted that their model can be validated with other ethnic groups. The current study offers the unique findings contributing to this idea in the South African setting. The study closed a research gap by validating OD and PV to enhance online shopping experience and e-shopping spending.

### 6.2 Practical implications

The study validated the importance of additional variables like OD, PS, PV, and PEoU to the original perspective of TAM in generating CT in online retailing. CT, OD, and PV are direct positive determinants of CL in B2C e-commerce. The relationships between PEoU, OD, PS, PV, and CL, are positively and indirectly mediated by CT in online retailing. In addition, OD and PV are variables that strengthen the trust and loyalty of customers with low online shopping experience and low e-shopping spending in the South African setting. This study's results differ from the findings in the previous research. For example, the current literature only shown the positive impact of product attributes on customer satisfaction [84,86,96], yet satisfaction strengthens CL when their levels of trust in the e-business are higher [30]. Furthermore, a direct positive and significant impact of PV on CL was confirmed by Ref. [111], who examined assortment (the variety of products or services a website offers), which had no direct effect on CT, but had a significant effect on CL [86]. could not find a significant impact of product portfolio on CL. This study shows that customers develop loyalty to an e-retailer that offers PV, and that the impact of OD of the products and PV are strong predictors for instilling CT and building CL, which are unique contributions.

This study offers key practical implications for managing online retailing. OD contributes the highest significant standardised estimate, which shows its power in generating CT and CL. Since customers prefer direct-to-home delivery [37], practitioners can improve their daily practice by providing real-time delivery information and return policies, reduce the delivery time, and ensure that the customer receives the order at an estimated arrival time, which could possibly enhance customer's confidence in the integrity and reliability of an e-retailer, and develop customer loyalty. For rural areas [11], suggested that e-retailers can use AI technologies together with third-party logistics to deliver products on time.

The key strategy for online retail practitioners is to include factors like OD, PS, PV, and PEoU within their regular practice to grow online shopping adoption and generate CT in online retailing. Because CT, OD, and PV are main factors that directly and positively impact CL in B2C e-commerce, it is likely that the practitioners could develop customers’ loyalty by gaining their trust, through delivering the product at the scheduled time, and if customers select from a wide product variety.

The relationships between PEoU, PS, OD, PV, and CL, are indirectly mediated by CT in online retailing, which is a unique contribution. As customers cannot touch and feel the product prior to purchasing online [3,8], ensuring the OD of the same ordered products and a wide variety will strongly establish CT and build CL, and thus maximise online retail sales. OD and PV are factors that build the trust and loyalty of customers with low online shopping experience and low e-shopping spending in the South African setting.

Due to the vulnerability of technology in security data breaches, improving PS of websites enhances CT, which grows the success of B2C e-commerce. Websites that present electronic security tools, i.e. data encryption, socket-layers, and third-party certificates will improve consumers' feeling of security in online shopping, and by declaring mutual security on websites, institution-based trust can be helpful to consumers; for example, an institutional “signature” enhances customers’ PS and the reliability of an e-retailer [76]. To sustain this advantage, e-retailers may need to promptly identify, contain, and reduce security breaches on their websites.

Because PV significantly improves CT, e-retailers must monitor inventory stock regularly to ensure that customers obtain the desired products, improve 3-D packaging in addition to ensuring delivery time, and inform a customer about any possible delay. Often, free shipping maximises the exchange rate and PV in the shopping cart [11].

Despite its low standard loading compared with other factors, PEoU significantly and positively generates CT. The ease of learning and becoming a skilled user of online retailing website technologies and interfaces is viewed as a successful strategy in designing easy to use technologies [53]. Considering the acceptance of technologies introduced in online retailing in South Africa, e-retailers that sell the variety of clothing and electronic gadgets must consider making ease of use part of strategic management in designing websites in order to improve CT. As a precursor of trust, designing website interfaces that are easy to use for online shoppers might help e-retailers to build customer trust in an emerging market such as South Africa.

Although PC might not be a significant factor affecting CT in online retailing context in South Africa, it suggests the need to consider a wider diversity of types of privacy-protective behaviours [124]. Perhaps research focusing on technology privacy assurances (e.g., encryption) rather than typical e-retailer-based measures (e.g., privacy policy) is needed [1] to measure and determine its impact on CT.

Considering that the relationship between OD and CL, and between PV and CL, are directly and indirectly mediated by CT, incorporating these factors in everyday activities could help increase customer trust and loyalty in online retailing. OD and PV are variables that strengthen the trust and loyalty of customers with low online shopping experience and low e-shopping spending in the South African setting. These practical implications show how this online retailing strategic approach could help online retailers to establish trust and build customer loyalty.

## 7 Conclusions, limitations and directions for future research

In general, this study suggests that the incorporating additional variables like OD, PS, PV, and PEoU to the original perspective of TAM generates CT in online retailing. CT, OD, and PV are important determinants of CL in B2C e-commerce. The positive relationships between OD and CL, and between PV and CL, are directly and indirectly mediated by CT in online retailing. The research also points to the OD and PV as variables that appear to strengthen the trust and loyalty of customers with low online shopping experience and low e-shopping spending in the South African setting. Although the study offered significant contributions to the technology acceptance theory, the model this study developed and validated in South Africa may be evaluated in other African countries, including several emerging economies, to generalise the reliability of its results and significance of its factors for other national societies. A longitudinal research method is needed. The measures used for experience and e-shopping spending consisted of a single items. Future research can expand this by investigating the moderation effects of these variables as a latent constructs, before generalisation can be made about their impact in online shopping in South Africa. PEoU had a lower standardised loading than the other factors, which shows that this factor needs more analysis to capture its significance in building online CT. This study did not measure the impact of perceived usefulness on CT, which offers limitations on the knowledge. Research that measures ease of use of technology system may adopt experimentation to observe if indeed it is easy for customers to use e-retailers websites. Research evaluating privacy assurances (e.g., encryption) rather than typical e-retailer-based measures (e.g., privacy policy) is needed [1]. Studies for SMEs in the townships of South Africa are needed to avoid a single look at bigger website brands such as Takealot.com, etc.

## Author contribution statement

Thabang Mofokeng: Conceived and designed the experiments; Performed the experiments; Analysed and interpreted the data; Contributed reagents, materials, analysis tools or data; Wrote the paper.

## Data availability statement

The data that has been used is confidential.

## Declaration of competing interest

The authors declare that they have no known competing financial interests or personal relationships that could have appeared to influence the work reported in this paper.
